# LC/UV determination of cefradine, cefuroxime, and cefotaxime in dairy milk, human serum and wastewater samples

**DOI:** 10.1186/2193-1801-2-575

**Published:** 2013-10-29

**Authors:** Tahira Qureshi, Najma Memon, Saima Q Memon, Kamran Abro, Syed Waliullah Shah

**Affiliations:** National Centre of Excellence in Analytical Chemistry, University of Sindh, Jamshoro, Sindh, Pakistan; Dr.M.A.Kazi Institute of Chemistry, University of Sindh, Jamshoro, Pakistan; Pakistan Council of Scientific and Industrial Research Laboratories Complex, Karachi, Pakistan; School Of Health Sciences, Universiti Sains Malaysia, Health Campus, Kota Bharu, 16150 Malaysia

**Keywords:** Pharmaceutically active compounds, High performance liquid chromatography, Hospital wastewater, Cephalosporins, Antibiotics, Milk

## Abstract

Cephalosporins type antibiotics are widely used to treat infectious diseases. Their determination is not only important in blood/serum of patients under treatment but also in diverse matrices like wastewaters, milk etc. as contaminant. Keeping in view the need, a new high performance liquid chromatographic method for the determination of three cephalosporins (cefradine, cefuroxime and cefotaxime) has been developed. Separation was performed on an ODS column with binary solvent elution of aqueous formic acid (0.05%) and methanol in the ratio of 45: 55 (v/v) at a flow rate of 1 mL min^-1^ and UV detection at 260 nm. Under optimised conditions, all three cephalosporins were baseline separated within 5 min. Linear responses for cefradine 5–20 μg mL^-1^, cefuroxime 0.5-15 μg mL^-1^ and cefotaxime 1.0-20 μg mL^-1^ were established. LOD of 0.05-0.25 μg mL^-1^ after preconcentration was achieved. The method was applied to serum samples of patients under treatment with these antibiotics and to screen the selected cephalosporins from hospital wastewater and milk samples. Moreover, method was applied to study stability of aqueous solutions and acid/base induced degradation of all three drugs.

## Introduction

Antibiotics constitute various groups of compounds which are used to treat bacterial infections. The main antibiotics used in human and veterinary medicine fall into following classes: β-lactams (β-LCs), tetracyclines (TCs), macrolides (MCs), aminoglycosides (AGs), amphenicols (AMPs), quinolones (Qs)/fluoroquinolones (FQs), sulfonamides (SAs), lincosamides (LCs), glycopeptides (GPs) and polyether ionophores (IPhs). From the group of β-lactams; penicillins and cephalosporins accounts for nearly 50-70% of antibiotics consumption in EU and USA etc. (Moreno-Bondi et al. 2009). Antibiotics are multi-group compounds and their determination in complex samples like environmental waters, milk and serum samples have been subject of interest to analytical and environmental chemistry. Several chromatographic assays, especially LC and CE coupled to MS or tandem MS are reported for screening of drugs in wastewater samples (Díaz-Cruz et al. 2007; Smyth and Rodriguez 2007; Batt et al. 2008; Bailón-Pérez et al. 2009; Díaz-Cruz et al. 2009; Moreno-Bondi et al. 2009; Seifrtová et al. 2009) whereas solid-phase extraction technique has remained method of choice for sample preparation (Benito-Peña et al. 2006; Batt et al. 2008; Samaras et al. 2010). Generally, biological fluid samples are treated using protein precipitation (Verdier et al. 2011) or solid phase extraction (Ohmori et al. 2011) whereas milk samples are simply diluted or injected directly (Cháfer-Pericás et al. 2010). However, application of LC/UV for complex matrices is limited to one report (Wang et al. 2011).

**Figure 1:**
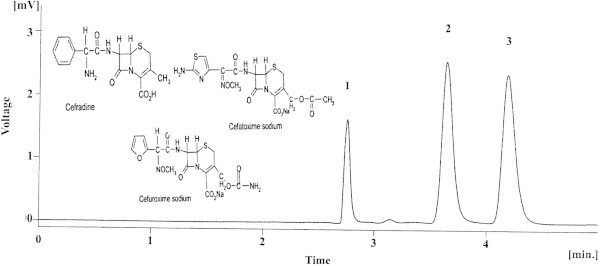
**Separation of three cephalosprins; cefradine (2.753), cefatoxime (3.740), cefuroxime (4.533) using 55%methanol+45% formicacid(0.05%) at flow rate of 1 mL min** ^**-1**^ **and λ**_**max**_ **260 nm along the structures of selected cephalosporins, cefradine, cefuroxime sodium and cefotaxime sodium.**

The conventional approach for screening of pharmaceuticals in environment/milk sample is based on identification of representative compound from each class of drugs (Araujo et al. 2011; Svanfelt and Kronberg 2011). For biological fluids, generalized methods depending on the type of cephalosporin administered using HPLC/UV, UPLC or HPLC/MS are reported (Denooz and Charlier 2008; Nemutlu et al. 2009; Wang and Li 2009; Ohmori et al. 2011). However, it must be noted that this approach does not target specific drugs used in selected location to screen water or milk samples. Therefore, it may lead to inadequate spatial screening of contaminants. Thus, it is of high importance that the screening should be based on a prior survey for the kind of drugs used in selected location. Also, the methods reported for biological samples cannot be applied without modifications to monitor blood/serum of patients under treatment with specific cephalosporin. Keeping in mind that hospitals are major stations for antibiotics usage, the doctors working in Liaquat University of Medical and Health Sciences, Jamshoro-Pakistan were consulted for the implication of antibiotics type. The most commonly employed antibiotics were found cephaolosporins, quinolines, sulfonamide and tetracycline whereas cephalosporins were predominantly employed of which cefradine (first generation), cefuroxime (second generation) and cefotaxime (third generation) are main drug compounds. The structure of three drugs is shown in Figure 1. The combination of drugs mentioned above is not reported earlier hence a chromatographic method using UV detection was developed and applied to real samples.

Since, these antibiotics are available in local market their presence in milk and other matrices cannot be ignored (Kantiani et al. 2009). Also, stability of cephalosporins in water is highly dependent on the time and the temperature (Gáspár et al. 2002).

Keeping in view the susceptibility of these three cephalosporins in diverse samples, chromatographic method was developed to determine three selected cephalosporins in various real world samples. Also the method was applied to study aqueous solution stability and induced hydrolysis (acid/base) of these drugs.

## Experimental

### Instrumentation

A Hitachi 6010 liquid chromatograph fitted with a Hitachi L-4200 variable wavelength UV–vis detector, a Rheodyne 7125 injector, and a Hibar^®^ C-18, 250 mm × 4.6 mm i.d. column by Merck (Germany) were used throughout the study. The CSW32 software (Data Apex) was used for data acquisition and integration.

An LC-MS (LCQ Advantage Max, Surveyor with quadruple and ion-trap system by Thermo (USA) comprising a Surveyor MS Pump and an autosampler with 20 μL sample volume was used for identification of the compounds. All the data was processed using the Pl. check software.

### Reagents and solutions

Methanol and formic acid were purchased from UK. All standards were from Germany and used as received.

All the three cephalosporin standard stock solutions were prepared in Millipore water (18 Ω) by dissolving the appropriate amount of each drug to get final concentration of 1000 μg mL^-1^. All the stock solutions were stored at 4°C and were found stable for one week. Working standards were prepared freshly in the mobile phase.

### LC and LC/MS conditions

Separation was carried out with a mobile phase composition of methanol and 0.05% formic acid (55:45) at a flow rate of 1.0 mL min^-1^. The sample injection volume was 20 μL, while UV detection was carried out at 260 nm.

LC/MS was operated on similar conditions as mentioned for LC-UV. MS was operated in ESI (positive ion) mode; needle voltage of 4.5 kV, probe temperature of 200°C, cone voltage of -29.6V, sheath gas flow rate of 53 arbitrary units, and auxiliary gas flow rate of 43.6 arbitrary units. Samples were run in SIM mode with three selected ions; 349.82, 455.50 and 447.8 for cefradine, cefatoxime and cefuroxime, respectively.

### Sample preparation

#### Hospital wastewaters

Hospital wastewater samples were collected from the out drain of Hospital of Liaqat University of Medical and Health Sciences, Jamshoro, Pakistan in the month of May 2011. Samples were filtered through 0.45 μm filter paper and a 200 mL aliquot was reduced to 10 mL using a rotary evaporator with vacuum pump V-700 (Switzerland) at reduced pressure keeping the temperature at 50°C.

Visiprep^®^ Solid-Phase extraction system fitted with mini vacuum pump (USA) was employed for clean-up. Oasis^®^ HLB cartridges 60 mg 3 mL^-1^ (Waters, USA) and C-18 50 mg 1mL^-1^ by Supelco (USA) were used. Cartridges were conditioned with 10 mL of 1:1 methanol/water and 10 mL of sample (preconcentrated by evaporation) was then passed sequentially through C-18 and HLB cartridges at flow rate of 1.0 mL min^-1^. The cartridge were then washed with 5 mL of Millipore water, air dried and the analytes were eluted with 10 mL of acetone from the Oasis HLB and with 10 mL of 55:45 ratio of MeOH/ aqueous formic acid (0.05%) from the C-18 cartridge. Both eluents were pooled together and evaporated to 2.0 mL under nitrogen stream. An aliquot was analyzed by the proposed HPLC method. After preconcentration and clean-up techniques cefatoxime, cefuroxime and cefradine can be detected down to 50, 50, and 100 ng mL^-1^ respectively in a mixture.

#### Milk samples

Five raw milk samples (500 mL each) were obtained from five different local dairies of Hyderabad, Pakistan. The samples were treated according to a previously reported method. Briefly, 2 mL of acetonitrile were added in 10 mL milk sample for protein precipitation. The mixture was then vortex mixed and centrifuged at 10000 rpm for 30 min. Further, sample was cleaned up using solid phase extraction with Oasis^®^ HLB cartridges 60 mg 3ml^-1^ by Waters (USA). The cartridge was conditioned with 2 mL of water, 2 mL of methanol and 2 mL of phosphate buffer (pH 5.5). Then the Supernatant of sample was loaded on the conditioned cartridge followed by washing with 3 mL water and air dried for 45 min. Finally, drugs were eluted with 8 mL acetone. The eluent was evaporated to dryness and made up with 2 mL of mobile phase (45:55 of 0.05% aqueous formic acid/methanol).

#### Serum samples

Venous blood sample of a group of four patients (aged 35–60 years) under treatment with cefradine, cefuroxime and cefotaxime were collected into a BD Vacutainer^®^ SST™ Tubes 2 h after the infusion of drug by intravenous injection. Samples were carried in ice packs by Temperatsure^©^ (USA) till transport to the laboratory then treated according to a reported method (Kinsella et al. 2009) with slight modifications. The blood samples (5 mL) were centrifuged at 3500 rpm for 25 min to separate the serum. In 2.5 mL of serum, 50 μL of acetonitrile was added and vortex mixed for two minutes. The solid phase extraction sample clean up was carried out in similar manner as described for wastewater samples. The final volume of sample before injection to HPLC was maintained at 2 mL with mobile phase. Also, the control samples spiked with standard of each drug were treated similarly and analyzed.

#### Stability / degradation studies

The solution stability of cefradine, cefuroxime and cefotaxime at room temperature was carried out by leaving the test solution (10 μg mL^-1^) in a tightly capped volumetric flask at room temperature (25 ± 3°C) for 30 h. The solution was assayed at 6 h intervals by drawing 20 μL solutions from the flask and injecting into HPLC.

The stability at elevated temperatures was examined in individually prepared solutions of 10 μg mL^-1^ of cefradine, cefuroxime and cefotaxime that were placed in water bath Julabo HC 5 (Germany) for 15 minutes at temperatures within the range of 30–70°C.

Base induced degradation was examined by adding 1 mL of 1.0 M NaOH to 10 mL of 10 μg mL^-1^ solution of each drug, following by heating on boiling water bath for 10 minutes. The solutions were neutralized with HCl (1.0 mol L^-1^) and 10 mL of mobile phase was added.

Degradation kinetics in aqueous solutions was studied by preparing 10 μg mL^-1^ solution of each drug and aliquot from this was injected with various time intervals. Decrease in peak area of each drug was plotted against time to plot degradation kinetics curve.

## Results and discussion

### HPLC method development

Separation of cefradine, cefuroxime and cefatoxime was initiated using RP-HPLC with formic acid in mobile phase as pH adjuster. Various parameters like; mobile phase composition, concentration of formic acid, flow rate, detection wavelength and solvent for sample were investigated. Organic modifier (methanol) was varied in the range 52–71% with neutral and acidified water (formic acid in the range of 0.05-0.1% was used). Mobile phase was found to induce pronounced effect on separation. Increase in methanol content increased the retention times while increasing aqueous content eventually merged the three components. Increase in retention time with increasing organic modifier may be due to methanolysis of cephalosporins at higher methanol content (Kinsella et al. 2009). Also basic pH was not good at resolving components due to ionization of compounds. Good separation in adequate time was achieved with 55% methanol and 45% formic acid (0.05%) modified aqueous phase. Increase in strength of formic acid increased the retention while separation remained unaffected.

Figure 1 shows the chromatogram obtained under optimized conditions, all the three compounds are baseline separated within 5 minutes. The theoretical plates were observed as; 9458 for cefradine, 6058 for cefuroxime and for cefotaxime 6457, where as the resolution were observed for peaks as, 2.33 and 6.92. Using optimized conditions linear calibration graph for cefradine 5–20 μg mL^-1^ (R^2^ = 0.979) 1.73 LOD and 5.76 LOQ, cefuroxime 0.5-15 μg mL^-1^ (R^2^ = 0.998) LOD 0.07 and 0.24 of LOQ and cefotaxime 1.0-20 μg mL^-1^ (R^2^ = 0.999) LOD 0.14 and LOQ 0.47 were established. The intra-day (n = 6) and inter-day precisions are shown in Table 1.

**Table 1:** **Intra-day and Inter-day precisions for cefradine, cefuroxime and cefotaxime**

Cephalosporin	Concentration	Intra-day	Inter-day**
	μg mL^-1^	% RSD *	% RSD
Cefradine	5	3.17	1.09
	15	0.50	1.30
	25	0.79	0.25
Cefuroxime			
	5	2.58	0.28
	15	0.44	0.37
	25	2.35	0.62
Cefotaxime			
	5	2.80	1.97
	15	1.83	0.62
	25	1.03	0.27

* Average from six replicate determinations, ** Average from five days determinations.

### Induced hydrolysis of cephalosporins

Cephalosporins undergo hydrolysis under various conditions like in the presence of metals, acid/base or enzymes (Deshpande et al. 2004; Seifrtová et al. 2009). However, the extent of hydrolysis for specific cephalosporin varies significantly which depends upon the reaction conditions and structure of the drug.

**Figure 2:**
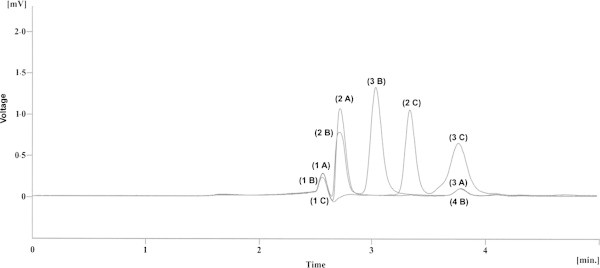
**Acid induced hydrolysis (1 M HCl) for 10 minutes at 70°C cefradine (A), cefuroxime (B) cefatoxime (C).**

In this study, hydrolysis was induced using HCl (1M) and NaOH (1M) for acid and base hydrolysis, respectively at elevated temperatures (70°C). Figure 2 shows the chromatogram of each drug after acid hydrolysis. Cefradine (A) decomposed 21%, cefuroxime 100% (B) and cefatoxime 92.8% (C) using induction time of 10 minutes. Moreover, cefradine was degraded into two more compounds; peak 1 A and 3 A while peak 2 A is parent compound whereas cefuroxime showed three additional peaks 1 B, 2 B and 3 B where as peak 4 B is parent compound and cefatoxime showed two additional peaks 1 C and 3 C, peak 2 C is parent compound.

Base hydrolysis completely decomposed all the drug compounds (98-100% degradation) as only one distorted peak was observed for cefradine and cefatoxime and many small peaks for cefuroxime with no peak at their corresponding retention times (chromatograms not shown).

**Figure 3:**
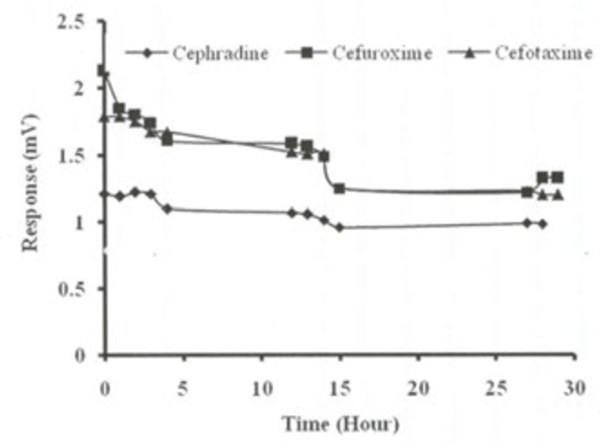
**The graphical views at room temperature aqueous solution stability of cefradine, cefuroxime and cefatoxime.**

The data suggests that base hydrolysis is fast and induce more rigorous conditions for selected cephalosporins. Figure 3 shows the degradation of three drugs in aqueous solutions at room temperature. 20% cefradine, 30% cefouroxime and cefatoxime degraded during 30 h. The degradation was found faster at initial five hours then slowed down and remained constant after 10 hour to 28 hours.

### Sample preconcentration and clean-up

Solid phase extraction is common technique to clean sample and preconcentrate pharmaceutical compounds. Various sorption materials; C-18, ENV+, HLB and other are reported whereas hydrophilic-lipophilic polymeric phases are reported for β-lactams (Puig et al. 2007) and widely accepted because of their capability to preconcentrate/clean-up a wide range of compounds. However, most of the methods have reported recoveries at higher concentrations (>/= 1 μg mL^-1^) while in real wastewaters lower μg or ng mL^-1^ ranges are usually observed. So, solutions containing low concentrations of three cephalosporins were prepared and 200 mL of each antibiotic was loaded onto HLB cartridges for preconcentration and eluted with various solvents as shown in Table 2. To achieve the better recoveries, other material (C-18) as single phase or in combination was also tried.

**Table 2:** **Recoveries of cefuroxime, cefatoxime and cefradine using SPE at various concentrations and with different elution solvents**

Drug	Conc. (ng mL^-1)^	Final volume	Adsorbent	Elution solvent	Recovery (%)
Drugs optimized individually					
Cefuroxime	10	2	HLB	80% MeOH	5
Cefatoxime	10	2	HLB	80% MeOH	24
Cefatoxime	10	2	HLB	100% MeOH	79
Cefuroxime	10	2	HLB	100% MeOH	47
Cefradine	20		C-18	55:45 MeOH:0.05% formic acid	90
Recovery using mixture of drugs					
Cefatoxime	50	2	HLB	100% MeOH	26
Cefatoxime	50	2	HLB	2 mL MeOH with 8 mL acetone	53
Cefatoxime	50	2	HLB	8 mL acetone	85
Cefuroxime	50	2	HLB	100% MeOH	29
Cefuroxime	50	2	HLB	8 mL acetne	73
Cefuroxime	50	2	HLB	8 mL acetone	77
Cefradine	50	2	HLB	100% MeOH	00
Cefradine	50	2	C-18	55:45 MeOH:0.05% formic acid	78
Recovery with HLB and C-18 in series with mixture of drugs					
Cefatoxime	50	2	Mixed mode	8 mL acetone	104
Cefuroxime	50	2	Mixed mode	8 mL acetone	44
Cefradine	100	2	Mixed mode	55:45 MeOH:0.05% formic acid	55
Recoveries at 1 μg mL^-1^ concentrations					
Cefatoxime		2	Mixed mode	8 mL acetone	95
Cefurxime		2	Mixed mode	8 mL acetone	83
Cefradine		2	Mixed mode	55:45 MeOH:0.05% formic acid	80

Adsorption of cephalosporins onto SPE materials and then recovery studies were initially carried out for single drug, and then all three drugs were loaded in mixture. The recoveries of three cephalosporins in low ng mL^-1^ range (10, 10 and 50 ng mL^-1^ for cefuroxime, cefatoxime and cefradine, respectively) using reverse-phase and HLB type cartridges.

Cefatoxime and cefuroxime showed recovery of 79% and 47% respectively when run as single compound while cefradine showed good recovery on C-18 with elution solvent using 55% MeOH and 45% aqueous formic acid. When all three drugs are simultaneously loaded onto sorbent materials and eluted with methanol and acetone or mixtures from HLB and MeOH + acidified water from C-18, the recovery varied appreciably; cefatoxime and cefuroxime showed even better recoveries using HLB cartridges and acetone as elution solvent as compared to methanol. However, cefradine better responded onto C-18 with acidified methanol. Since, same sorbent material did not show good response to all three drugs, both the materials were connected in series; HLB followed by C-18 and 200 mL containing 50–100 ng mL^-1^ of drugs was passed through cartridges, air dried and then drugs were eluted separately using acetone for HLB and acidified methanol for C-18. Both eluates were pooled; solvents were evaporated under nitrogen stream and then volume was made up to 4 mL with mobile phase and injected onto HPLC system for recovery studies. Cefatoxime showed very good recovery but cefuroxime and cefradine proved to be poorly recovered. The reason may be low concentrations of drugs which renders the favourable interactions of molecules for good adsorption hence results in losses during recovery studies. On the other hand, recoveries at 1 μg mL^-1^ were higher and acceptable. Evaporation of solvent is one of the techniques which can be used to enrich the low concentration or bringing concentrations of trace compounds in the ranges suitable for SPE. However, evaporation of water from samples by boiling-off may degrade cephalosporins so possible degradation of three drugs was studied at various temperatures (Table 3). At 50°C, all the drugs were fairly stable and this temperature was kept constant and pressure was reduced enough to remove the water by evaporation.

**Table 3:** **Stability of drugs at various temperatures**

Temperature (°C)	Cefradine (%)	Cefuroxime (%)	Cefatoxime (%)
30	99.86	97.00	94.74
40	92.21	93.87	90.24
50	90.98	91.65	90.19
60	83.79	85.34	69.55
70	80.72	82.72	66.52

Clean-up step was not necessary for standard solutions of drugs but for samples where matrix also preconcentrate along with analyte and may interfere in chromatographic assay procedure. Therefore, wastewater samples were preconcentrated and cleaned-up by evaporation followed by solid phase extraction using optimized extraction protocol whereas milk and serum samples were as processed with reported methods (Denooz and Charlier 2008; Junza et al. 2011).

### Application of method

#### Synthetic and hospital wastewater samples

The optimized method for preconcentration and chromatographic separation was employed to determine three cephalosporins in synthetic wastewater and hospital wastewater. Composition of synthetic wastewater is given in (Boeije 1999). Since, hospital wastewater contains many unknown compounds; the samples were run using same liquid chromatographic procedure with mass spectrometric detection. Also, spiked synthetic wastewater was run parallel to hospital wastewater to check the performance of assay procedure. LC/MS procedure is given in experimental. Presence of each compound was confirmed by matching retention time with that of standard and molecular ion peak. Cefradine molecular ion peak of 349.82 at t_R_ 3.24 minutes, cefotaxime 455.50 at t_R_ 3.53 minutes and cefuroxime 447.8 (M + Na) at t_R_ 3.9 minutes were used for confirmation. Good recoveries (77.8 – 112.5%) were observed for spiked synthetic wastewater containing of 50 μg L^-1^ (cefuroxime and cefotaxime) and for cefradine (100 μg L^-1^) but none of the drug was detected in hospital wastewater. The data is consistent with previous reports and may be explained because of degradation of drug compounds by complex matrix effects, sunlight and possibly adsorption on soil (Bundgaard and Larsen 1983; Deshpande et al. 2004). However, one recent report has demonstrated the identification of cephalosporin in wastewaters (Wang and Li 2009).

#### Serum samples

Serum samples from patients under treatment with selected drugs were analysed using developed chromatographic procedure. Cefradine was detected in higher concentration (1.810 ± 0.002 μg mL^-1^) as compared to cefatoxime (0.400 ± 0.002 μg mL^-1^) and cefuroxime (0.410 ± 0.002 μg mL^-1^). This may be due to relatively higher doses of cefradine (1 g twice a day) injected to patients as compared to cefuroxime and cefatoxime (0.25 g twice a day).

#### Milk samples

Antibiotics are used as feed additives in order to enhance feed efficiency. Contaminated animal products are available for consumption as a result of either illegal use of β-lactams or non-compliance of producers with existing animal-treatment protocols (withdrawal times). Therefore, residues of these substances may enter the food chain .The milk samples collected from local diaries were analyzed to check the presence of the cephalosporins. The cefradine and cefotaxime were not found in milk samples, where as one of the samples was found contaminated with cefuroxime. The amount of cefuroxime found in contaminated milk sample was 7.38 ± 0.02 μg L^-1^ which was confirmed by spiking the sample with 5 μg mL ^-1^ , then in increments of 2 μg mL ^-1^ of cefuroxime for three times.

## Conclusion

An LC/UV method for determination of low μg L^-1^ ranges of cefradine, cefuroxime and cefatoxime in diverse matrices was developed. Room temperature aqueous of cefuroxime and ceftoxime shows that both have similar stability while cefradine is more stable than other two drugs. Base hydrolysis in all cases was found faster than acid hydrolysis for all three drugs. Wastewater was not found contaminated with selected cephalosporins at method’s detection limit. The method was successfully applied to determine cephalosporins in milk samples and patients’ serum under treatment with these drugs. It could be concluded that simple RP-LC method can be used to determine these cephalosporins using appropriate sample clean up.
